# DNA Microarray-Based Global Gene Expression Profiling in Human Amniotic Epithelial Cells Predicts the Potential of Microalgae-Derived Squalene for the Nervous System and Metabolic Health

**DOI:** 10.3390/biomedicines10010048

**Published:** 2021-12-27

**Authors:** Farhana Ferdousi, Kinji Furuya, Kazunori Sasaki, Yun-Wen Zheng, Tatsuya Oda, Hiroko Isoda

**Affiliations:** 1 Alliance for Research on the Mediterranean and North Africa (ARENA), University of Tsukuba, Tsukuba 305-8572, Japan; ferdousi.farhana.fn@u.tsukuba.ac.jp (F.F.); sasaki-kazu@aist.go.jp (K.S.); 2 Faculty of Life and Environmental Sciences, University of Tsukuba, Tsukuba 305-8575, Japan; 3 AIST-University of Tsukuba Open Innovation Laboratory for Food and Medicinal Resource Engineering (FoodMed-OIL), University of Tsukuba, Tsukuba 305-8572, Japan; ywzheng@md.tsukuba.ac.jp (Y.-W.Z.); tatoda@md.tsukuba.ac.jp (T.O.); 4 Department of Gastrointestinal and Hepato-Biliary-Pancreatic Surgery, Faculty of Medicine, University of Tsukuba, Tsukuba 305-0005, Japan; kfuruya@md.tsukuba.ac.jp

**Keywords:** human amniotic epithelial cell, DNA microarray analysis, squalene, *Aurantiochytrium* Sp., functional enrichment, gene-disease association, gene ontology, nervous system diseases, metabolic diseases

## Abstract

In recent years, perinatal stem cells, such as human amniotic epithelial cells (hAECs), have attracted increasing interest as a novel tool of stem cell-based high-throughput drug screening. In the present study, we investigated the bioactivities of squalene (SQ) derived from ethanol extract (99.5%) of a microalgae *Aurantiochytrium* Sp. (EEA-SQ) in hAECs using whole-genome DNA microarray analysis. Tissue enrichment analysis showed that the brain was the most significantly enriched tissue by the differentially expressed genes (DEGs) between EEA-SQ-treated and control hAECs. Further gene set enrichment analysis and tissue-specific functional analysis revealed biological functions related to nervous system development, neurogenesis, and neurotransmitter modulation. Several adipose tissue-specific genes and functions were also enriched. Gene-disease association analysis showed nervous system-, metabolic-, and immune-related diseases were enriched. Altogether, our study suggests the potential health benefits of microalgae-derived SQ and we would further encourage investigation in EEA-SQ and its derivatives as potential therapeutics for nervous system- and metabolism-related diseases.

## 1. Introduction

In recent years, stem cells have been used extensively in high-throughput drug screening, from identifying target compounds to evaluating compounds at the preclinical stage [1,2,3]. In addition, the recent identification and isolation of human pluripotent adult stem cell lines from cord blood, adipose tissue, and bone marrow have offered opportunities in drug discovery and increased confidence in the mechanism of action of new targets [4]. However, although stem cell-based methods reduce the period of product development and rate of attrition of new therapeutics, their invasive extraction procedures, expensive cell reprogramming and maintenance procedures, and ethical constraints are the primary impediments to considering them as practical sources for drug screening [5].

In this regard, perinatal stem cells, such as human amniotic epithelial cells (hAECs), are gaining interest as a novel source of pluripotent stem cells (PSCs) with significant advantages over other types of stem cell. hAECs are derived from the term placenta after delivery, pose no ethical concerns, and do not require invasive harvesting procedures. hAECs are not tumorigenic and have immunomodulatory as well as weak immunogenic properties [6,7,8,9]. Most importantly, hAECs show ESC-like multilineage differentiation potential and can differentiate into cells of all three germ layers, such as neural cells from the ectodermal origin, liver, and pancreas from the endodermal origin, and bone and adipose cells from the mesodermal origin [7,10,11,12,13,14]. hAECs, which are isolated from the adherent subpopulations of passaged primary cells and cultured in a 3D spheroid environment, express higher stemness properties [15,16]. In our previous studies, we have examined the gene expression profiling of several natural compounds in hAECs to explore their novel bioactivities. Applying whole-genome transcriptome analysis in hAECs, we explored the therapeutic potential of verbenalin for Alzheimer’s disease [17], isorhamnetin for cardiac hypertrophy and fibrosis [18], and cyanidin-3-O-glucoside for adipose tissue dysfunction [19]. We have also reported the differentiation-inducing potential of rosmarinic acid, 3,4,5-tri-O-caffeoylquinic acid, and isorhamnetin in hAECs [15,20,21].

In the present study, we investigated the bioactivities of squalene (SQ) derived from a microalgae *Aurantiochytrium* sp. in hAECs using whole-genome transcriptome analysis. SQ is a natural triterpene and a biosynthesized precursor to human steroids. It is naturally found in high concentrations in shark liver. SQ is widely used in the cosmetic and pharmaceutical industries because of its immune functions and antioxidant activities. Additionally, studies showed that SQ modulates fatty acid metabolism, decreases serum cholesterol levels, and suppresses tumor proliferation [22,23]. However, the high costs, as well as environmental concerns have prompted a search for alternative sources of SQ [24,25]. Some microorganisms are attracting interest for their ability to synthesize large amounts of SQ. *Aurantiochytrium* is a thraustochytrid, a heterotrophic marine oleaginous microorganism [26,27]. At the University of Tsukuba, a researcher group identified an *Aurantiochytrium* strain in the Okinawa prefecture of Japan that can accumulate very high SQ (198 mg/g) [28]. Our previous studies have reported that ethanol extract of *Aurantiochytrium* 18W-13a (EEA) has anti-inflammatory [29,30], antidepressant-like [31], and neuroprotective effects [32]. This SQ rich EEA (EEA-SQ) showed better neuroprotective and neuroproliferative effects compared to SQ only and the n-hexane layer of EEA (squalene-rich fraction) [32]. The current study aimed to explore the detailed biological functions of EEA-SQ in hAECs using hAEC as a drug screening tool and to identify novel bioactivities of EEA-SQ.

## 2. Materials and Methods

### 2.1. Preparation of Squalene-Rich Ethanol Extract of Aurantiochytrium 18W-13a (EEA-SQ)

Professor Makoto Watanabe (Algae Biomass and Energy System R&D Center, University of Tsukuba, Japan) generously provided us with the dried powder of *Aurantiochytrium* sp. The dried powder of the algae was stored at −80 °C and was extracted at room temperature and in the dark using 99.5% ethanol. The container of the algae mixture was shaken once daily. After 2 weeks, the liquid fraction was obtained and then microfiltered through a 0.22-μm membrane filter. The EEA-SQ liquid fraction was concentrated using a SpeedVac (Thermo Fisher Scientific, Japan) and then dissolved in Eagle’s minimum essential serum-free medium through sonication (OPTI-MEM; Gibco, Japan). hAECs were treated with EEA-SQ at a 20 µg/mL concentration following our previous studies [31,32] in human neuroblastoma cells (SHSY5Y cells) and neurospheres of mouse neural progenitor cells. EEA-SQ is safe to use on human cells in general. Cytotoxicity of EEA was observed at the concentration of 1 mg/mL in murine macrophage RAW264 cells [29].

### 2.2. Human Amniotic Epithelial Cells (hAECs) Extraction, Culture, and 3D Spheroid Formation

Detailed procedures of hAEC extraction and culture conditions have been explained elsewhere [15,16]. Briefly, the amnion was separated from the chorion and was washed with Hank’s Basic Salt Solution—calcium and magnesium free (CMF-HBSS; 200 mL) to remove blot clots before cutting it using surgical scissors. Cut pieces of the amnion were placed into 50 mL conical tubes with a pre-digestion buffer (CMF-HBSS, 0.5mM EGTA, Wako Pure Chemical Industries Ltd., Osaka, Japan, 20 mL) and were incubated at 37 °C for 10 min. Then the membrane was incubated in 0.05% trypsin-EDTA (Thermo Fisher Scientific, Waltham, MA, USA) at 37 °C for 40 min and placed on ice after incubation. Finally, Dulbecco’s Modified Eagle Medium (DMEM, 10% FBS, 1% Penn-Strep, two volumes) was added and centrifuged at 200 g at 4 °C for 10 min to detach the epithelial cells from the membrane. The supernatant was discarded and the pellet was resuspended in 10 mL of DMEM. A 100 µm strainer (Merck EMD, Darmstadt, Germany) was used to remove small tissue aggregates, and clots from the cell suspension and the primary AECs were isolated. On 0.4% trypan blue dye (Dojindo Laboratories, Kumamoto, Japan) cell viability of AECs was found to be >90%. hAECs were maintained in the PromoCell Placental Epithelial Cell Basal Medium (PromoCell, C-26140).

The hAECs were cultured in the Corning^®^ Elplasia^®^ 3D culture plates (Elplasia^TM^, Kuraray Co., Ltd., Tokyo, Japan) using Lipidure^TM^ coating (NOF Corporation, Tokyo, Japan) as described previously [15]. Spheroids were formed by seeding 1 × 10^6^ hAECs into each well of the 24-well plate. The initial culture was maintained for 24 h.

### 2.3. hAEC Treatment

hAECs were treated with EEA-SQ at the concentration of 20 µg/mL for 7 days following the previous protocols of hAEC treatment [15,17,19,20]. The medium was changed with EEA-SQ every 48 h (total three times during the 7-day treatment period) following the manufacturer’s protocol (ElplasiaTM, Kuraray Co., Ltd., Tokyo, Japan). Control samples were in the basal medium (PromoCell Placental Epithelial Cell Basal Medium), which was also changed every 48 h. Finally, RNA samples were collected for three biological replicates of each EEA-SQ-treated and control hAECs on day 7 and prepared for microarray analysis as reported in our previous studies [15,17,19,20]. We attempted time-dependent microarray analysis on hAEC using different natural compounds. We found that day 7 to day 10 were the best time points to study the early biological events induced by specific compounds. Before day 7, the effects of the compounds cannot be observed. After day 10, hAECs begin to determine their fate choice, and therefore, not suitable for functionality screening of the compounds.

### 2.4. RNA Extraction and Quantification

We used the Isogen kit (Nippon Gene Co., Ltd., Tokyo, Japan) to extract total RNA. RNA quantity and quality were determined using the NanoDrop 2000 spectrophotometer (ThermoScientific, Tokyo, Japan).

### 2.5. Microarray Gene Expression

We performed microarray analysis with Affymetrix’s GeneAtlas^®^ System (Affymetrix Inc., Santa Clara, CA, USA). Firstly, total RNA samples were prepared from the three replicates of each control and EEA-SQ-treated hAEC sample (250 ng per RNA sample). Next, the amplified and biotin-labeled complementary RNA samples (cRNAs) were generated using the GeneChip 3′ IVT PLUS Reagent Kit (Affymetrix Inc., Santa Clara, CA, USA). Then the cRNA samples were fragmented and labeled and then prepared for hybridization using the GeneAtlas^®^ Hybridization, Wash, and Stain Kit for 3’ IVT Array Strips. Finally, the human genome array strips (HG-U219) were hybridized for 16 h at 45 °C on the GeneAtlas^®^ Hybridization Station, washed and stained with the GeneAtlas^®^ Fluidics Station, and scanned with the GeneAtlas^®^ Imaging Station.

### 2.6. Microarray Data Processing and Analysis

The raw image data were normalized following the robust multichip average (RMA) algorithm using the Expression Console Software (Affymetrix, Japan, URL: http://www.affymetrix.com, accessed on 30 November 2017). Subsequent gene expression analysis was carried out using freely available software Transcriptome Analysis Console (TAC) version 4 (Thermofisher Inc., Tokyo, Japan). Genes with a linear fold change >1.1 and *p*-value < 0.05 (one-way between-subjects ANOVA) were considered as differentially expressed genes (DEGs). The tissue specificity of the DEGs was determined using the Tissue-Specific Expression Analysis (TSEA) web tool (URL: http://genetics.wustl.edu/jdlab/tsea/, accessed on 14 October 2021). Tissue enrichment of the DEGs was identified by Fisher’s exact test with the Benjamini–Hochberg correction and was expressed as the ‘Specificity Index’ thresholds (pSI) of varying stringency from 0.05 to 0.001. Genes with pSI < 0.0001 were the most specific genes enriched in a particular tissue [33,34].

Gene set enrichment analysis (GSEA) was carried out to identify significantly enriched hallmark gene sets and KEGG pathways using the Molecular Signatures Database (MSigDB) ver. 7.4 of GSEA online software (URL: https://software.broadinstitute.org/gsea/index.jsp/, accessed on 22 October 2021) [35].

HumanBase public database (URL: https://hb.flatironinstitute.org, accessed on 22 October 2021) and its analytical tool ‘Module’ was used to identify functionally clustered modules by the DEGs based on shared k-nearest-neighbors (SKNN) and the Louvain community-finding algorithm [36]. The SKNN-based strategy follows several steps to identify highly specific edges and to link genes that are highly likely to be part of the same functional cluster. Firstly, a user-selected tissue-specific network subset is created that contains the user-provided genes (nodes) and each edge between nodes, which are associated with a certain weight. Based on the original weight of the edges, a new weight is calculated that is equal to the number of k nearest neighbors. Then the top 5% of the edges, according to new edge weights, are selected to create the shared-nearest-neighbor tissue-specific network. Finally, the Louvain algorithm is applied to cluster this network into distinct functional modules. In order to stabilize the clustering, the Louvain algorithm is run 100 times and the score of cluster co-membership of each pair of genes/nodes equal to the fraction of times (out of 100) the pair was assigned to the same cluster is calculated. Genes with a co-membership score ≥0.9 are assigned to clusters. The resulting modules are tested for functional enrichment using genes annotated to Gene Ontology (GO) biological process (BP) terms and are presented with their resulting Q-value. The Q-value of each term within the modules is calculated using the one-sided Fisher’s exact tests followed by Benjamini–Hochberg corrections. Similarly, tissue-specific functional modules were identified for both up and downregulated DEGs. Additionally, tissue and biological process-specific gene networks, tissue expression, and BP terms were retrieved for the top 19 up and downregulated DEGs.

Curated gene-disease association data were retrieved from the Comparative Toxicogenomics Database (CTD) (URL: http://ctdbase.org//, accessed on 22 October 2021) [37]. CTD contains two types of gene–disease associations—‘curated’ and ‘inferred’. The curated associations are either extracted from the published literature or derived from the OMIM database using the NCBI Gene database. Inferred associations are based on CTD–curated chemical–gene interactions. We identified the diseases that are statistically enriched by the DEGs using the Set Analyzer tool that performs set-based enrichment analyses and pathway generation for user-provided genes. The significance of enrichment was calculated by the hypergeometric distribution adjusted by the Bonferroni method. MyGeneVenn tool was used to create Venn diagrams to compare our gene list to genes with direct relationships to specific disease terms (filtered for exact terms only). We checked for both inferred and curated gene-disease associations. Finally, genes with direct evidence of disease association were identified and presented according to their inference score. Inference scores are calculated for all inferred relationships and represent the similarity degree between the CTD chemical–gene–disease networks and a similar scale-free random network.

All data supporting the findings of this study are included within the article or its Supplementary Files. We have deposited the microarray data into the Gene Expression Omnibus (GEO) under the Accession Number: GSE188411.

### 2.7. Ethics Approval

The protocol was approved by the Ethical Review Committee of the University of Tsukuba Hospital (protocol approval code: H27-58, date: 10 July 2015). Informed written consent was obtained from the placenta donors.

## 3. Results

### 3.1. Characteristics of Gene Expression Profiling in EEA-SQ-Treated hAECs

**Figure 1:**
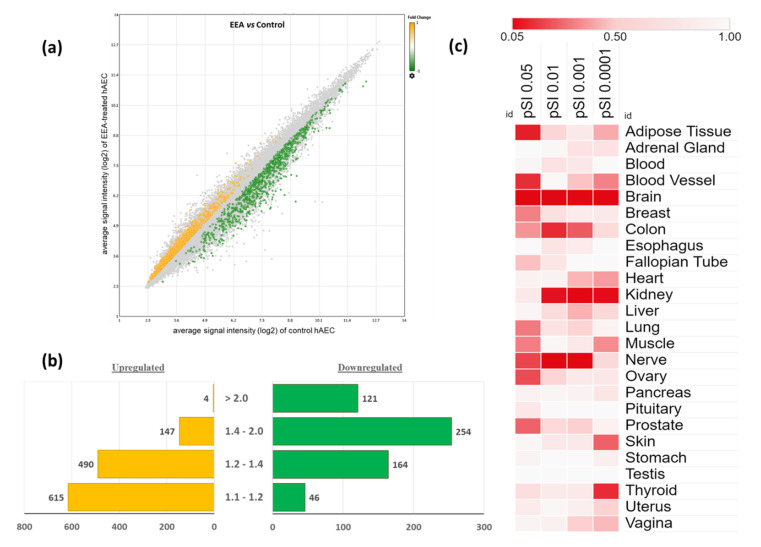
Microarray gene expression profile of squalene-rich ethanol extract of *Aurantiochytrium* 18W-13a (EEA-SQ)-treated human amniotic epithelial cells (hAECs). (**a**) Scatter plot showing the differentially expressed genes (DEGs) between EEA-SQ-treated and control hAECs. The X-axis displays the average signal intensity (log2) of genes in EEA-SQ-treated hAECs and Y-axis corresponds to the average signal intensity (log2) of genes in control hAECs. Up and downregulated DEGs are presented in the yellow and green dots, respectively. (**b**) Bar graph displays the number of DEGs and distribution of fold changes. (**c**) Heat map showing the significance and specificity of the expressions of the DEGs across 25 tissues. Enrichment analysis was conducted using the Tissue-Specific Expression Analysis (TSEA) tool (http://genetics.wustl.edu/jdlab/tsea//, accessed on 14 October 2021). Heat map was generated on Morpheus tool (https://software.broadinstitute.org/morpheus//, accessed on 28 October 2021).

A total of 49,386 probe sets and 20,083 unique genes were identified on HG-U219 array strips. At day 7, 1841 genes were differentially expressed in the EEA-SQ treated hAECs compared to control hAEC (fold change > 1.1 and *p*-value < 0.05). Among them, 1256 DEGs were upregulated and 584 were downregulated. The scatter plot shows the signal intensities of the DEGs in EEA-SQ-treated and control hAECs (Figure 1a). Up and downregulated DEGs are shown in yellow and green dots, respectively. Bar graphs show the distribution of fold changes of up and downregulated DEGs (Figure 1b).

As reported in our previous studies, we have also compared the day 7 EEA-SQ-treated and control data sets with the day 0 control hAECs. Consistent with our previous findings [15,17,20], a comparison between day 0 and day 7 control hAECs (untreated cells) showed only early biological events related to cell differentiation, cell cycle arrest, and regulation of cell proliferation (data not presented). In contrast, a comparison between day 0 control and day 7 EEA-SQ-treated hAECs showed similar, but more significant, effects observed when we compared between day 7 EEA-SQ-treated and untreated (day 7) control hAECs. Therefore, in the present study, we restricted our analysis to only day 7 hAECs (treated vs. control) to avoid any exaggeration of the findings.

### 3.2. Tissue-Specific Enrichment Analysis of the Differentially Expressed Genes (DEGs)

Enrichment analysis of the expressions of the DEGs across 25 tissues was performed using the TSEA web tool (Figure 1c). Among the DEGs (n = 1841), 1703 genes were found to be expressed in at least one tissue. We found that the brain was the most significantly enriched tissue by the DEGs in all specificity index thresholds (pSI) varying from 0.005 to 0.0001. A total of 229 DEGs were brain-specific. Among them, 31 had the highest specificity threshold (pSI < 0.0001, *p*-value = 0.002), 35 had pSI < 0.001 (*p*-value = 0.001), 64 had pSI < 0.01 (*p*-value = 0.003), and 99 had pSI < 0.05 (*p*-value = 0.007). Table 1 shows the overlapped genes with pSI < 0.0001 and their functions. Brain-specific DEGs are involved in chemical synaptic transmission, axon development, neurotransmitter secretion, response to wounding, axon ensheathment, locomotion, negative regulation of neuron apoptotic process, astrocyte development, glutamate secretion, long-term synaptic potentiation, and positive regulation of neurogenesis.

The next significantly enriched tissue was the kidney. Several kidney-specific (pSI < 0.001) solute carrier genes were identified, responsible for ion transport. The skin and thyroid were also significantly enriched at pSI threshold < 0.0001. Skin-specific DEGs regulate several important biological processes, such as skin development, fatty acid metabolic process, keratinocyte differentiation, lipoxygenase pathway, linoleic acid metabolic process, regulation of water loss via skin. Thyroid-specific DEGs are involved in thyroxine biosynthesis, G protein-coupled receptors (GPCR) downstream signaling, and lipoprotein metabolism.

Adipose tissue and blood vessels were also significantly enriched; however, most of the overlapped genes were in a low specificity threshold (pSI < 0.05). Only two adipose tissue-specific genes had the highest stringency (pSI < 0.0001), which are involved in negative regulation of the lipid catabolic process, and insulin-stimulated glucose uptake in adipocytes. On the other hand, several DEGs were overlapped with testis-specific gene sets (pSI < 0.0001) but did not reach the statistical significance level (*p*-value = 1.0).

### 3.3. Gene Set Enrichment Analysis

**Figure 2:**
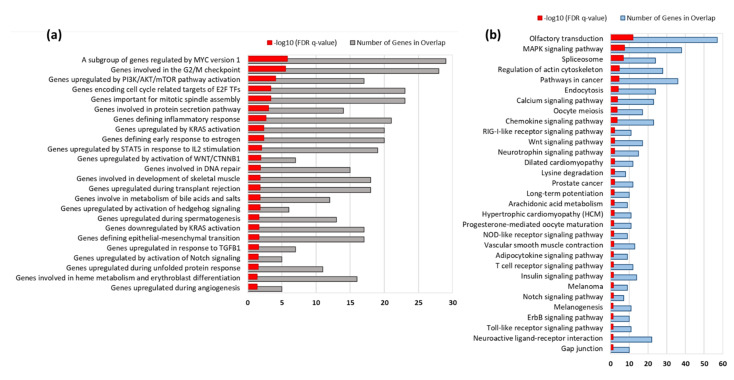
(**a**) Bar graph showing the top enriched Hallmark gene sets. (**b**) Bar graph showing the significant KEGG pathways. Gene set enrichment analysis was conducted using MsigDB (https://www.gsea-msigdb.org/gsea/index.jsp/, accessed on 22 October 2021). Significance was considered at false discovery rate (FDR) *q*-value < 0.05.

Next, we identified the top enriched hallmark gene sets using the MsigDB (Figure 2a). Significance was measured as the false discovery rate (FDR *q*-value < 0.05; an analog of hypergeometric *p*-value following Benjamini–Hochberg correction. Hallmark gene sets ‘summarize and represent specific, well-defined biological states or processes and display coherent expression’. There are 50 hallmark gene sets that condense information from over 4000 sets and, therefore, that have reduced noise and redundancy.

We found that EEA-SQ significantly enriched several cell cycle-related genes sets, such as ‘Genes involved in the G2/M checkpoint’, ‘Genes encoding cell cycle-related targets of E2F TFs’ as well as ‘Genes defining epithelial-mesenchymal transition’. EEA-SQ also significantly enriched gene sets of signaling pathways regulate the core cellular processes of proliferation, differentiation, and migration, such as ‘Genes upregulated by activation of WNT/CTNNB1’, ‘Genes upregulated by activation of hedgehog signaling’, and ‘Genes upregulated by activation of Notch signaling’. Several inflammatory pathway-related gene sets were also regulated by EEA-SQ, such as ‘Genes defining inflammatory response’, ‘Genes upregulated by PI3K/AKT/mTOR pathway activation’, ‘Genes upregulated by STAT5 in response to IL2 stimulation’, ‘Genes upregulated in response to TGFB1′, and ‘Genes downregulated by KRAS activation’.

### 3.4. KEGG Pathways Enriched by EEA-SQ

Significantly enriched KEGG pathways (FDR *q*-value < 0.05) were identified on the MSigDB web tool (Figure 2b). Along with Wnt and Notch signaling pathways, EEA-SQ significantly enriched MAPK, calcium, chemokine, RIG-I-like receptor, neurotrophin, NOD-like receptor, ErbB, and Toll-like receptor signaling pathways. Interestingly, EEA-SQ also regulated adipocytokine and insulin signaling pathways. Other important pathways include long-term potentiation, melanoma, neuroactive ligand-receptor interaction, and vascular smooth muscle contraction.

### 3.5. Detection of Gene Clusters and Functional Module

**Figure 3:**
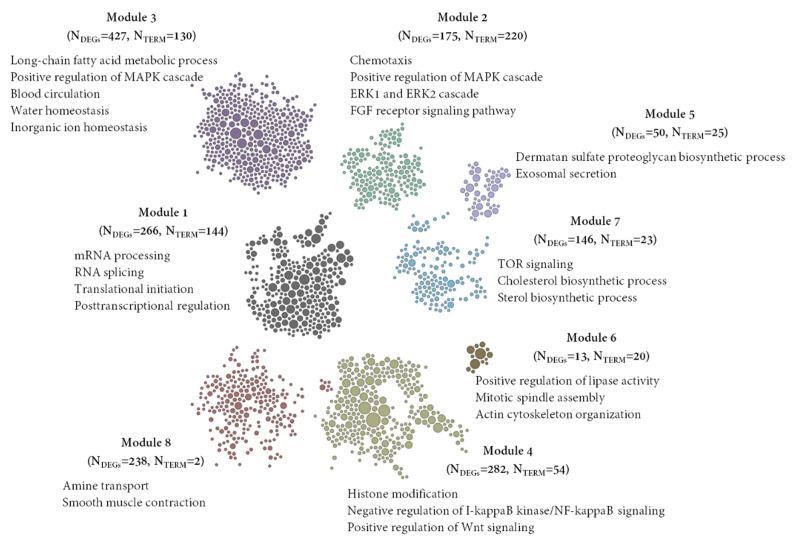
Functionally clustered modules by the DEGs based on shared k-nearest-neighbors and the Louvain community-finding algorithm using the HumanBase public database (https://hb.flatironinstitute.org//, accessed on 22 October 2021). Significantly enriched Gene Ontology biological process (GOBP) terms of each module are presented. Significance was calculated using Fisher’s exact tests followed by Benjamini–Hochberg corrections. N_TERMs_ = Number of enriched GOBP terms in each module, N_DEGs_ = number of differentially expressed genes in each module.

We detected functionally clustered genes based on SKNN and the Louvain community-finding algorithm using the HumanBase public database, enabling network-based functional interpretation of the DEGs. Each module consists of tightly connected genes with a co-membership score ≥ 0.9. For each module, GOBP terms were detected for functional enrichment (Figure 3).

A total of 1620 DEGs were assigned to a module and eight distinct functional modules were detected. Module 1 (M1) consisted of 266 functionally connected DEGs that enriched 144 BP terms. Top enriched BP terms in M1 were regulation of mRNA processing (GO:0050684), and RNA splicing (GO:0008380). DEGs in M2 (*n* = 175) enriched chemotaxis (GO:0006935), positive regulation of MAPK cascade (GO:0043410), and positive regulation of ERK1 and ERK2 cascade (GO:0070374). DEGs in M2 also enriched BP terms related to the development and morphogenesis of the renal tubule, urogenital system, blood vessels, and cardiovascular system (Supplementary File S1). DEGs in M3 (*n* = 427) significantly enriched regulation of body fluid levels (GO:0050878), long-chain fatty acid metabolic process (GO:0001676), water homeostasis (GO:0030104), and regulation of blood circulation (GO:1903522). Global functional modules and tissue-specific functional modules, and enriched BP terms in each module, have been described in Supplementary File S1.

### 3.6. Tissue-Specific Biological Functions

**Figure 4:**
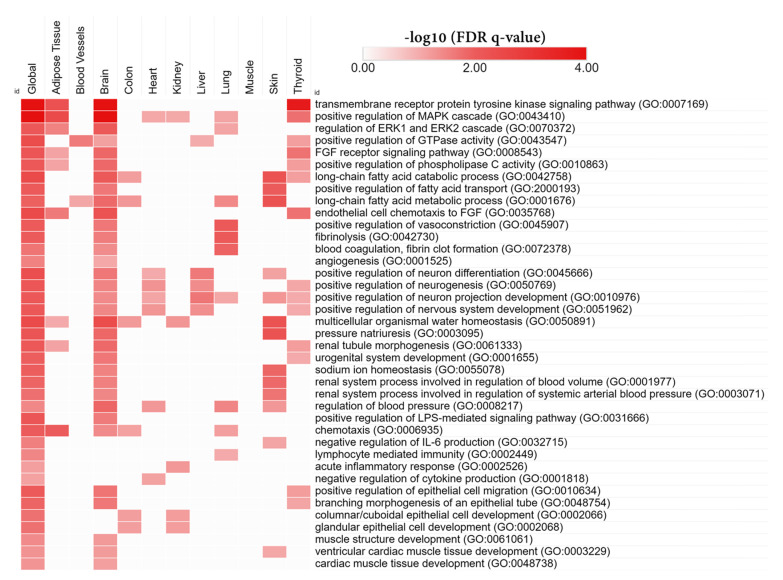
Tissue-specific network-based functional analysis of the upregulated DEGs.

**Figure 5:**
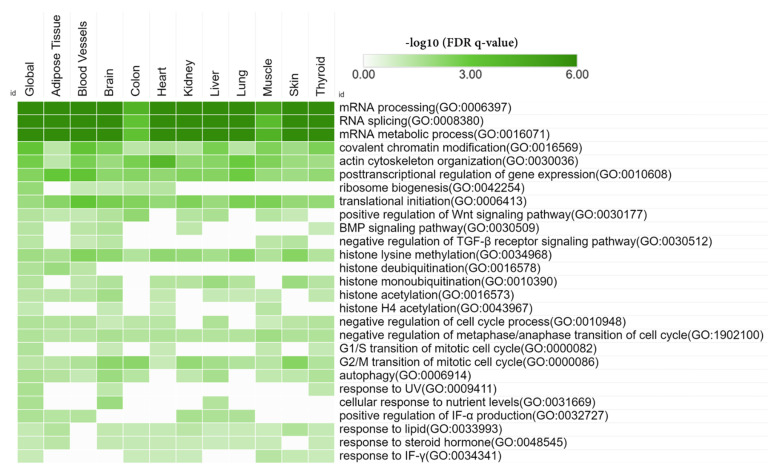
Tissue-specific network-based functional analysis of the downregulated DEGs.

We performed tissue-specific network-based functional interpretation of the upregulated (Figure 4) and downregulated (Figure 5) DEGs using the HumanBase web tool. We identified functional modules specific to adipose tissue, blood vessels, brain, colon, heart, kidney, liver, lung, muscle, skin, and thyroid, considering significantly enriched tissues by the DEGs (Figure 1c).

We found that upregulated DEGs showed mostly brain-specific functional enrichment (Figure 4). Except for the inflammatory pathways (negative regulation interleukin-6 production (GO:0032715), lymphocyte-mediated immunity (GO:0002449), and acute inflammatory response (GO:0002526)), almost all the global BP terms were also brain-specific. Other important brain-specific BPs were positive regulation of neuron differentiation (GO:0045666), positive regulation of neurogenesis (GO:0050769), positive regulation of neuron projection development (GO:0010976), and positive regulation of nervous system development (GO:0051962).

There were also adipose tissue, skin and thyroid-specific functional enrichment by the upregulated DEGs. Adipose tissue-specific top BP terms were the transmembrane receptor protein tyrosine kinase signaling pathway (GO:0007169), positive regulation of MAPK cascade (GO:0043410), regulation of ERK1 and ERK2 cascade (GO:0070372), positive regulation of protein serine/threonine kinase activity (GO:0071902) fibroblast growth factor receptor signaling pathway (GO:0008543), positive regulation of phospholipase C activity (GO:0010863), regulation of lipid biosynthetic process (GO:0046890), chemotaxis (GO:0006935), and response to wounding (GO:0009611). Skin-specific top BP terms were the long-chain fatty acid catabolic process (GO:0042758), positive regulation of fatty acid transport (GO:2000193), long-chain fatty acid metabolic process (GO:0001676), positive regulation of lipid localization (GO:1905954), blood circulation (GO:0008015), multicellular organismal water homeostasis (GO:0050891), and negative regulation of interleukin-6 production (GO:0032715). On the other hand, thyroid-specific BP terms were the transmembrane receptor protein tyrosine kinase signaling pathway (GO:0007169), positive regulation of MAPK cascade (GO:0043410), positive regulation of protein serine/threonine kinase activity (GO:0071902), positive regulation of cell migration (GO:0030335), positive regulation of cell motility (GO:2000147), cellular response to growth factor stimulus (GO:0071363), positive regulation of GTPase activity (GO:0043547), peptidyl-tyrosine phosphorylation (GO:0018108), peptidyl-tyrosine modification (GO:0018212), fibroblast growth factor receptor signaling pathway (GO:0008543), positive regulation of phospholipase C activity (GO:0010863), protein autophosphorylation (GO:0046777), endothelial cell chemotaxis to fibroblast growth factor (GO:0035768), platelet-derived growth factor receptor signaling pathway (GO:0048008), blood circulation (GO:0008015). Kidney-specific significant BPs were columnar/cuboidal epithelial cell development (GO:0002066), glandular epithelial cell development (GO:0002068), enteroendocrine cell differentiation (GO:0035883), and glandular epithelial cell differentiation (GO:0002067).

Downregulated DEGs significantly enriched RNA processing (mRNA processing (GO:0006397), RNA splicing (GO:0008380), ribosome biogenesis (GO:0042254), and translational initiation (GO:0006413)), histone modification (histone lysine methylation (GO:0034968), histone methylation (GO:0016571), histone deubiquitination (GO:0016578), histone monoubiquitination (GO:0010390), and histone acetylation (GO:0016573)), and cell cycle (negative regulation of metaphase/anaphase transition of cell cycle (GO:1902100), G1/S transition of mitotic cell cycle (GO:0000082), and G2/M transition of mitotic cell cycle(GO:0000086))-related BP terms. Downregulated DEGs also enriched some important BP terms such as BMP signaling pathway (GO:0030509), negative regulation of TGF beta receptor signaling pathway (GO:0030512), and positive regulation of canonical Wnt signaling pathway (GO:0090263). Autophagy (GO:0006914), positive regulation of interferon-alpha production (GO:0032727), and response to interferon-gamma (GO:0034341) terms were also enriched by downregulated DEGs. However, downregulated DEGs did not show any particular tissue-specific functional enrichment (Figure 4). Tissue-specific modules for up and downregulated DEGs are provided in the Supplementary File S2.

### 3.7. Functional Gene Networks

**Figure 6:**
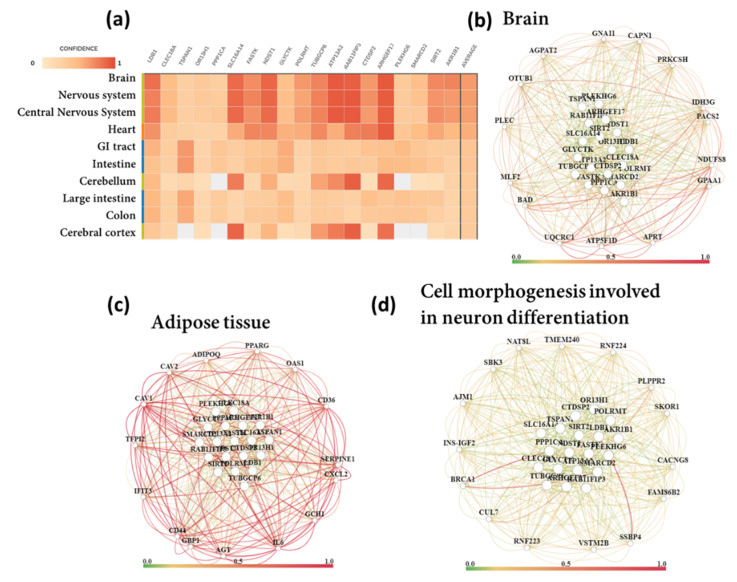
Tissue and biological process-specific functional gene network analysis for top 19 upregulated DEGs. (**a**) Tissue expression, (**b**) brain-specific functional network, (**c**) adipose tissue-specific functional network, (**d**) cell morphogenesis involved in neuron differentiation-specific functional network.

**Figure 7:**
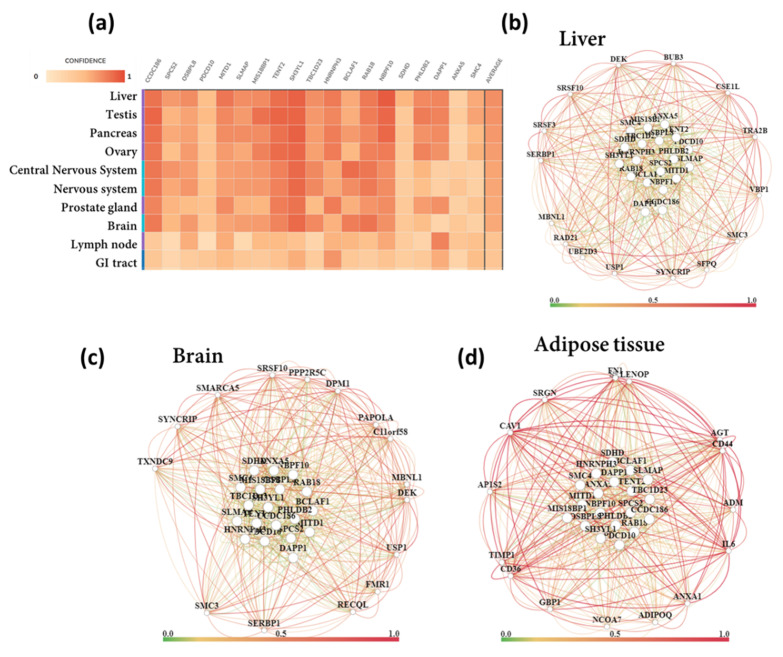
Tissue and biological process-specific functional gene network analysis for the top 19 downregulated DEGs. (**a**) Tissue expression, (**b**) liver-specific functional network, (**c**) brain-specific functional network, (**d**) adipose tissue-specific functional network.

We performed tissue and biological process-specific gene networks for top 19 upregulated (Figure 6) and downregulated (Figure 7) DEGs. Among the top upregulated DEGs, Rho guanine nucleotide exchange factor 17 (*ARHGEF17*), RAB11 family interacting protein 3 (*RAB11FIP3*), ATPase type 13A2 (*ATP13A2*), N-deacetylase/N-sulfotransferase (heparan glucosaminyl) 1 (*NDST1*), solute carrier family 16, member 14 (*SLC16A14*), LIM domain binding 1 (*LDB1*) are predominantly expressed in brain and nervous tissue (Figure 6). Therefore, we tested for brain-specific functional networks. Top brain-specific functionally related genes were ATP synthase F1 subunit delta (*ATP5F1D*), BCL2 associated agonist of cell death (*BAD*), NADH: ubiquinone oxidoreductase core subunit S8 (*NDUFS8*), OTU deubiquitinase (*OTUB1*), calpain 1 (*CAPN1*), glycosylphosphatidylinositol anchor attachment 1 (*GPAA1*), phosphofurin acidic cluster sorting protein 2 (*PACS2*), protein kinase C substrate 80K-H (*PRKCSH*), myeloid leukemia factor 2 (*MLF2*), adenine phosphoribosyltransferase (*APRT*), 1-acylglycerol-3-phosphate O-acyltransferase 2 (*AGPAT2*), isocitrate dehydrogenase 3 (NAD(+)) gamma (*IDH3G*), G protein subunit alpha 11 (*GNA11*), and plectin (*PLEC*). However, no biological processes and diseases were represented in the brain-specific network of the top 20 upregulated DEGs. Therefore, we further detected the functional network of neurons differentiation-specific (BP term: cell morphogenesis involved in neuron differentiation). The top connected genes for neuron differentiation were BRCA1 DNA repair associated (*BRCA1*), single-stranded DNA binding protein 4 (*SSBP4*), V-set and transmembrane domain-containing 2B (*VSTM2B*), phospholipid phosphatase related 2 (*PLPPR2*), transmembrane protein 240 (*TMEM240*), and cullin 7 (*CUL 7*).

We also detected adipose tissue and renal tubule-specific functional networks for the top upregulated DEGs. The top connected renal tubule-specific genes were forkhead box C1 (*FOXC1*), aquaporin 1 (*AQP1*), bone morphogenetic protein 7 (*BMP7*), QKI KH domain-containing RNA binding (*QKI*), solute carrier organic anion transporter family member 3A1 (*SLCO3A1*), BCL2 associated athanogene 1 (*BAG1*), paired box 2 (*PAX2*), forkhead box J1 (*FOXJ1*), and collagen type XVIII alpha 1 chain (*COL18A1*). The top enriched functions (BPs) represented in the renal tubule-specific network were metanephros development, mesonephros development, cellular response to salt stress, and cellular hyperosmotic response. Adipose tissue-specific functionally related genes were caveolin 1 (*CAV1*), interleukin 6 (*IL6*), CD36 molecule (*CD36*), angiotensinogen (*AGT*), peroxisome proliferator activated receptor gamma (*PPARG*), 2’-5’-oligoadenylate synthetase 1 (*OAS1*), serpin family E member 1 (*SERPINE1*), caveolin 2 (*CAV2*), C-X-C motif chemokine ligand 2 (*CXCL2*), adiponectin C1Q and collagen domain-containing (*ADIPOQ*), activating transcription factor 3 (*ATF3*), GTP cyclohydrolase 1 (*GCH1*), and signal transducer and activator of transcription 1 (*STAT1*). The top enriched BPs represented in the adipose tissue-specific network were response to bacterium, macrophage-derived foam cell differentiation, low-density lipoprotein particle receptor biosynthetic process, and lipoprotein transport.

On the other hand, the top 19 downregulated DEGs were diffusely expressed in liver, pancreas as well as in brain and nervous tissue (Figure 7). We detected liver, brain and adipose tissue-specific interactions. Several liver-specific interconnected genes were identified, such as ubiquitin-specific peptidase 1 (*USP1*), synaptotagmin binding cytoplasmic RNA interacting protein (*SYNCRIP*), VHL binding protein 1 (*VBP1*), serine and arginine-rich splicing factor 10 (*SRSF10*), RAD21 cohesin complex component (*RAD21*), and SERPINE1 mRNA binding protein 1 (*SERBP1*); however, top enriched BPs represented in the network were mRNA metabolic process and splicing. Brain-specific functionally related genes were poly(A) polymerase alpha (*PAPOLA*), *SRSF10*, *SYNCRIP*, *SERBP1*, and *USP1*; nevertheless, no BP enrichment was detected within the network.

Interestingly, adipose tissue-specific interactions were broadly similar for both top downregulated and top upregulated DEGs. The top downregulated DEGs were functionally interconnected to (adipose tissue-specific) *CAV1*, *CD36*, *IL6*, *ADIPOQ*, and *AGT*. Other adipose tissue-specific functionally connected genes were serglycin (SRGN), selenoprotein P (*SELENOP*), adaptor related protein complex 1 subunit sigma 2 (*AP1S2*), nuclear receptor coactivator 7 (*NCOA7*), annexin A1 (*ANXA1*), TIMP metallopeptidase inhibitor 1 (*TIMP1*), and CD44 molecule (*CD44*). The top enriched functions represented within the adipose tissue-specific network were response to wounding, negative regulation of cytokine secretion, low-density lipoprotein particle clearance, ERK1 and ERK2 cascade, and intrinsic apoptotic signaling pathway. The lists of top up and downregulated DEGs and their functions are presented in Table 2 and Table 3.

### 3.8. Gene–Disease Associations

**Figure 8:**
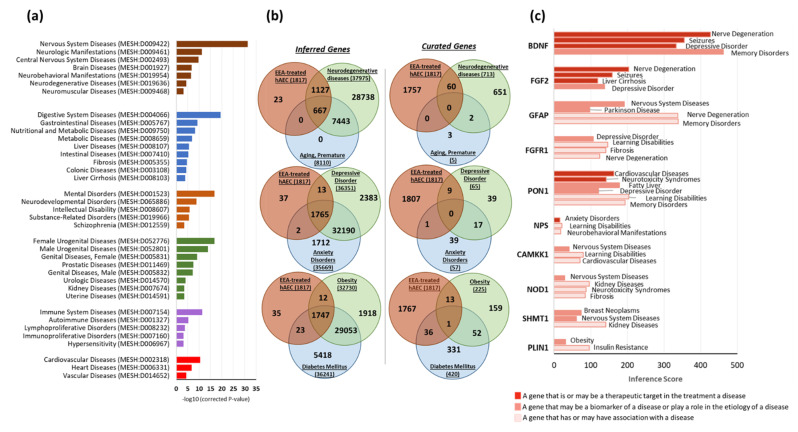
Curated gene-disease association. (**a**) Bar graph showing statistically enriched diseases terms (MESH) by the DEGs using the Set Analyzer tool of the Comparative Toxicogenomics Database (CTD). The significance of enrichment is calculated by the hypergeometric distribution and adjusted for multiple testing using the Bonferroni method. (**b**) Comparison of gene list (in EEA-SQ-treated hAECs) with direct relationships to specific disease terms for both inferred and curated gene-disease associations. MyGeneVenn tool of CTD data base was used to create Venn diagrams. (**c**) Bar graph showing the inference score of selected genes with direct evidence of disease association.

We detected significantly enriched diseases by the DEGs using the publicly available database CTD. Diseases (MEDIC terms) with corrected *p*-value < 0.0001 (hypergeometric distribution adjusted by Bonferroni method) were considered statistically enriched. We found that nervous system-related diseases, such as neuromuscular diseases (MESH: D009468), neurodegenerative diseases (MESH: D019636), neurobehavioral manifestations (MESH: D019954), brain diseases (MESH: D001927), central nervous system diseases (MESH: D002493), and neurologic manifestations (MESH: D009461) were the most significantly enriched (Figure 8a). Digestive system diseases, such as metabolic diseases (MESH: D008659), nutritional and metabolic diseases (MESH: D009750) as well as liver diseases (MESH: D008107), fibrosis (MESH: D005355), and liver cirrhosis (MESH: D008103), were also significantly enriched. Mental and neurodevelopmental disorders, immune system diseases, and cardiovascular diseases were also among the significantly enriched diseases (Figure 8a).

Next, we identified the genes in our gene list that have inferred or curated association to nervous system diseases, namely neurodegenerative diseases, premature aging, depression and anxiety, and adipose tissue dysfunctions, such as obesity and diabetes mellitus (DM). Curated gene-disease associations are determined by CTD and OMIM curation and represent a more robust association compared to the inferred gene–disease associations, which are obtained via CTD-curated chemical–gene interactions. We selected the genes that have only direct relationships to the disease terms and filtered out the hierarchical relationship genes. We found that 60 DEGs from our gene list were directly associated with neurodegenerative diseases, 9 DEGs to depressive orders, 1 DEG to anxiety disorders, 14 DEGs to obesity, and 37 DEGs to DM (Figure 8b).

From curated gene–disease associations, we identified several genes that may be responsible for the mechanism of action of EEA-SQ. Next, we examined the inference score of those genes to select the DEGs with specific disease associations. The inference score represents the similarity degree between CTD chemical–gene–disease networks and scale-free random networks. The higher the score, the more likely the inference network has atypical connectivity. Brain-derived neurotrophic factor (*BDNF*), fibroblast growth factor 2 (*FGF2*), fibroblast growth factor receptor 1 (*FGFR1*), paraoxonase 1 (*PON1*), neuropeptide S (*NPS*), glial fibrillary acidic protein (*GFAP*), calcium/calmodulin-dependent protein kinase kinase 1, alpha (*CAMKK1*), nucleotide-binding oligomerization domain containing 1 (*NOD1*), serine hydroxymethyltransferase 1 (soluble) (*SHMT1*), and perilipin 1 (*PLIN1*) were the most important DEGs that have a curated association to a disease (either therapeutic target or marker of a disease) or inferred association with a very high inference score (Figure 8c). Their expression profile in EEA-SQ-treated hAECs, BPs, and disease association are described in Supplementary File S3.

## 4. Discussion

In the present study, we have performed integrated transcriptome analysis of EEA-SQ-treated hAECs. Our findings suggest the prospects of EEA-SQ in nervous system diseases and adipose tissue dysfunctions.

Natural resources are chemically diverse and are able to modulate several biological functions simultaneously. DNA microarrays have become a necessary tool to investigate the mechanisms of action and drug development of natural bioactive compounds [38,39]. While the conventional gene expression profiling are optimized for single gene analysis, microarrays generate gene expression data on a genome-wide scale and therefore permit systematic approaches to the biological discovery of regulatory mechanisms and biochemical pathways [40]. However, even employing high throughput tools like DNA microarrays may not improve the success rate for drug discovery and development as the essential biological elements of drug-target interactions are mostly lost in the monolayer cell cultures [41]. In the present study, we used 3D hAEC spheroid to understand better the effect of EEA-SQ in a physiologically relevant context.

Previously we showed that different natural compounds could direct the differentiation of hAECs towards multiple cell lineages. A caffeoylquinic acid derivative-TCQA [20] and a caffeic acid ester-rosmarinic acid [15] enhanced neural cell differentiation in hAECs, whereas a flavonol, isorhamnetin, induced hepatic-lineage specific [21] and an anthocyanin CY3G induced adipocyte differentiation [19] in hAECs. We noticed that the bioactivities of natural compounds could broadly be predicted from the enriched cell types by differentially expressed genes. In the present study, we found that the DEGs by EEA-SQ treatment in hAECs could significantly enrich brain tissue, i.e., EEA-SQ primarily regulated brain-specific gene expression. Additionally, adipose tissue, blood vessel, colon, kidney, skin, thyroid tissues were enriched (Figure 1c). However, when we performed tissue-specific functional enrichment analysis, only brain, adipose tissue, and skin-specific biologic functions (BPs) remained relevant (Figure 4 and Figure 5). The hallmark gene sets identified cell cycle-related functions, and core pathways involved in cell proliferation and differentiation, such as Wnt, Notch, and hedgehog signaling (Figure 2a); however, KEGG pathway analysis could additionally identify the MAPK pathway, chemokine, calcium and neurotrophin signaling pathways, long-term potentiation and neuroactive ligand–receptor interaction (Figure 2b). When we performed tissue-specific functional network analysis, it became evident that EEA-SQ mainly significantly regulated nervous system development, neuron differentiation, neurogenesis, and neuron projection development. Brain-specific DEGs are also involved in neurotransmitter secretion and antiapoptotic and anti-inflammatory functions in the brain (Table 1). When we limited our analysis to only the top regulated DEGs (highest fold change), we found that the top upregulated DEGs are primarily expressed in the brain and are functionally related to multiple brain-specific and neuron-differentiation-specific genes (Figure 7). For example, the top upregulated DEGs are functionally associated with *BRCA1*, which is highly expressed in adult neurogenic areas and regulates distinct apoptotic functions in neural progenitors [42]. Further gene–disease association analysis could identify some target genes of EEA-SQ for its therapeutic potential in nervous system-related diseases, such as *BDNF*, *FGF2*, *GFAP*, *PON1*, and *NPS*. BDNF is a neurotrophic factor that promotes neuronal survival, growth, and maturation [43,44,45]. FGF2, a multi-functional growth factor, is expressed in the subventricular zone (SVZ) and the subgranular zone (SGZ) of the hippocampal dentate gyrus and regulates neural stem and progenitor cells and, therefore, has been implicated in adult neurogenesis [46]. *GFAP* is expressed in the astrocytes and is involved in cell communication and the functioning of the blood-brain barrier in the central nervous system. On the other hand, *PON1* is associated with neurobehavioral development [47,48]. A neuropeptide NPS increases the excitatory transmitter glutamate secretion and produces anxiolytic-like effects by reducing fear responses [49]. All these findings align with our previous studies on EEA-SQ that reported antidepressant-like, neuroprotective, and cognition-enhancing effects of EEA-SQ in vivo [31,32].

Additionally, some critical adipose tissue-specific functions were observed in EEA-SQ-treated hAECs. Significantly enriched KEGG pathways include adipocytokine and insulin signaling pathways and tissue-specific functional analysis reveals positive regulation of MAPK cascade. Furthermore, functional network analysis of the top DEGs identified several functionally connected genes in adipose tissue, including *PPARG*, *SERPINE1*, *CD36*, *CAV1*, *CAV2*, and *ADIPOQ*. PPARG is the key regulator of adipogenesis, is involved in fatty acid metabolism and triglyceride storage, and suppresses the production of inflammatory mediators in macrophages [50]. SERPINE1 is a unique insulin-sensitizing adipocytokine and, like PPARG, is implicated in a wide range of metabolic diseases, including DM, obesity, and fatty liver diseases [51]. CD36 is a marker of human adipocyte progenitors [52], CAV1 and CAV2 are important regulators of adipose tissue homeostasis [53], and ADIPOQ is observed exclusively in mature fat cells [54]. Indeed, the top enriched BPs represented in the adipose tissue-specific network included antiinflammatory, lipoprotein particle receptor biosynthetic process, and lipoprotein transport as well as lipid droplet formation. Lipid droplets in the form of foam cells derived from macrophages are a hallmark for the progression of atherosclerosis [55], which explains the enrichment of cardiovascular diseases by the DEGs. We also found that EEA-SQ significantly upregulated the expression of PLIN1, a regulator of lipid storage and lipolysis [56]. It is worth noting that in our previous study we reported that SQ and its derivative improved the metabolism of adipocytes differentiated from diabetic adipose-derived stem cells by enhancing energy homeostasis and insulin sensitivity and also prevented excessive lipogenesis [57].

Previously, we have reported anti-inflammatory effects of *Aurantiochytrium* strains in murine macrophages [29,30]. In hAECs, EEA-SQ also significantly enriched antiinflammatory hallmark gene sets and KEGG pathways. However, functional analysis could not detect any tissue-specific anti-inflammatory function. Therefore, we assume that EEA-SQ may regulate the systemic anti-inflammatory function instead of the tissue-specific mechanism of inflammation resolution.

Additionally, our TSEA analysis showed that skin is one of the significantly enriched tissues (Figure 1c). Functional module analysis (Figure 3) also revealed enrichment of the dermatan sulfate proteoglycan biosynthetic process (GO:0050651) and exosomal secretion (GO:1990182). It has been reported that alteration of dermatan sulfate proteoglycan biosynthesis in the fibroblasts of skin results in severe skin structure disruption [58,59]. Exosomes are made of a lipid bilayer structure containing lipids, nucleic acids and proteins and are secreted by most cell types. Previous studies reported the efficacy of exosomes in a number of skin defects, including skin aging, skin inflammation, atopic dermatitis, and pigmentation [60,61,62,63]. Also, tissue-specific functional analysis showed that the long-chain fatty acid metabolic process, water homeostasis, and antiinflammatory functions were significantly enriched in skin (Figure 4). SQ is very popular in cosmetics products as it maintains skin’s moisture barrier and hydration [25]. Our findings suggest that EEA-SQ may also have similar beneficial properties for skin.

Although our study explores multiple beneficial prospects of EEA-SQ, our results at present are observational in nature at the whole-genome transcriptome level. However, some of our observations are consistent with our previous animal-model studies [31,32] that reported antidepressant and neuroprotective effects of EEA-SQ. Further in vitro study focusing on the time- and dose-dependent effects of EEA-SQ on hAEC morphology and phenotypes are required to confirm the potential functionalities of EEA-SQ observed in the present study.

## 5. Conclusions

Altogether, our findings suggest the potential health benefits of EEA-SQ, notably in neurological and neurodegenerative diseases and metabolic disorders with adipose tissue dysfunction. Thus, our work may promote interest in and encourage investigation of EEA-SQ and its derivatives as potential therapeutics for nervous system- and metabolism-related diseases.

## Figures and Tables

**Table 1:** List of tissue-specific (pSI < 0.0001) genes and their function.

Tissue	Overlapped Genes (pSI Threshold 0.0001)	Functions
Adipose Tissue	*PLIN1, TUSC5*	Negative regulation of lipid catabolic process, insulin-stimulated glucose uptake in adipocytes
Adrenal Gland	*KCNK3, GSTA3*	potassium channel activity, Glutathione metabolism
Blood	*none*	
Blood Vessel	*ACAN*	Integrin Pathway, extracellular signal-regulated kinase (ERK) Signaling, mitogen-activated protein kinase (MAPK) Signaling
Brain	*HTR5A, BARHL1, GAP43, MBP, SNAP25, HTR2C, PLP1, RPH3A, DIRAS2, GABRA6, GFAP, CACNG3, OPCML, GAD2, RESP18, SLC4A10, SLCO1A2, JPH3, LRTM2, STMN2, LIX1, SLC1A2, NEFL, SLC6A7, ZIC5, OLFM3, PRKCG, GRM4, CACNG7, HS3ST4, OTP*	Chemical synaptic transmission, axon development, neurotransmitter secretion, response to wounding, axon ensheathment, locomotion, negative regulation of neuron apoptotic process, astrocyte development, glutamate secretion, long-term synaptic potentiation, positive regulation of neurogenesis
Breast	*FDCSP*	Secreted protein in follicular dendritic cells
Colon	*LRRC31, MS4A12, CDH17, FXYD3, CEACAM7, CEACAM5, MUC13*	Homophilic cell adhesion via plasma membrane adhesion molecules, integral component of membrane
Esophagus	*none*	
Fallopian Tube	*none*	
Heart	*BMP10, MYBPC3*	Ventricular cardiac muscle tissue morphogenesis, heart contraction, regulation of striated muscle contraction, blood circulation
Kidney	*SLC5A12, SLC12A1, PAX2, SLC13A1, FOXI1, SLC34A1, ATP6V0D2*	Inorganic anion transmembrane transport, Na-ion transmembrane transporter activity
Liver	*PLG, FGB, FGG, UGT1A1*	Negative regulation of blood coagulation, negative regulation of extrinsic apoptotic signaling pathway, response to Interleukin-1 (IL-1)
Lung	*SFTPA2*	Involved in respiratory gaseous exchange and toll-like receptor signaling pathway
Muscle	*MYH2, ASB12*	Enables microfilament motor activity, p21-activated kinase (PAK) Pathway, ubiquitin protein ligase activity, Innate Immune System
Nerve	*SLC25A48, OR6B2*	Integral component of membrane, signal transduction, involved in G protein-coupled receptor (GPCR) signaling pathway
Ovary	*WFIKKN2*	Negative regulation of Transforming growth factor-beta (TGF- β) receptor signaling pathway
Pancreas	*RBPJL*	Development and maintenance of the exocrine pancreas, Notch Signaling Pathway
Pituitary	*none*	
Prostate	*KLK2*	Prostate-specific antigenproduction
Skin	*LCE1C, ALOXE3, ALOX12B, LCE2B, LCE1F, CA6, PLA2G2F, KRT74, KRT82, KRT75, CYP4F8, FAM83C*	Skin development, fatty acid metabolic process, keratinocyte differentiation, lipoxygenase pathway, linoleic acid metabolic process, regulation of water loss via skin
Stomach	*GIF*	Transportation and absorption of the vital micronutrient vitamin B12
Testis	*RNF151, CTCFL, RNASE11, RNF17, DCAF4L2, C7ORF72, EFCAB3, SSX2B, FAM170A, C10ORF53, APOBEC4, MAGEC1, SIRPD, MAEL, UBQLN3, RFX4, DAZL, DEFB121, SLCO6A1, PABPC3, C5ORF60, OR8B12, SLC36A3, PRSS54, TKTL1, SYCP1, OPN5, VWA3B*	Spermatogenesis, DNA methylation involved in gamete generation, male gamete generation
Thyroid	*IYD, TSHR, LIPG, ZNF804B, SLC26A4*	Thyroxine biosynthesis, GPCR downstream signalling, Lipoprotein metabolism
Uterus	*PGR*	
Vagina	*TMPRSS11A, SERPINB13*	

**Table 2:** Top significantly upregulated DEGs and their biological function.

Gene Symbol	Description	Fold Change	*p*-Value	Biological Process *
*CLEC18A*	C-type lectin domain family 18, member A	2.53	0.026	
*TSPAN1*	tetraspanin 1	2.48	0.039	Regulation of vesicle-mediated transport, positive regulation of endocytosis
*OR13H1*	olfactory receptor, family 13, subfamily H, member 1	2.43	0.048	
*PPP1CA*	protein phosphatase 1, catalytic subunit, alpha isozyme	2.06	0.023	Circadian rhythm, positive regulation of ubiquitin-protein transferase activity, regulation of immune response, regulation of ERK1 and ERK2 cascade, positive regulation of MAPK cascade
*SLC16A14*	solute carrier family 16, member 14	1.98	0.007	Negative regulation of protein modification process
*LDB1*	LIM domain binding 1	1.9	0.042	Epigenetic regulation of gene expression, regulation of DNA binding transcription factor activity, positive regulation of cell differentiation, regulation of mitotic cell cycle
*FASTK*	Fas-activated serine/threonine kinase	1.85	0.004	Regulation of RNA splicing, Endoplasmic-reticulum-associated protein degradation (ERAD) pathway
*NDST1*	N-deacetylase/N-sulfotransferase (heparan glucosaminyl) 1	1.84	0.014	Cellular carbohydrate biosynthetic process, glycoprotein biosynthetic process
*GLYCTK*	glycerate kinase	1.83	0.044	Regulation of DNA binding transcription factor activity
*POLRMT*	polymerase (RNA) mitochondrial (DNA directed)	1.8	0.049	Mitochondrial transcription, RNA methylation, ribosome biogenesis
*TUBGCP6*	tubulin, gamma complex associated protein 6	1.8	0.027	Microtubule polymerization
*ATP13A2*	ATPase type 13A2	1.79	0.047	Cellular response to oxidative stress, cellular calcium ion homeostasis, regulation of macroautophagy, positive regulation of Notch signaling pathway, regulation of neurogenesis
*RAB11FIP3*	RAB11 family interacting protein 3 (class II)	1.78	0.028	Cell division, negative regulation of adiponectin secretion
*CTDSP2*	CTD small phosphatase 2	1.76	0.039	Protein dephosphorylation, positive regulation of DNA binding transcription factor activity, steroid hormone mediated signaling pathway
*ARHGEF17*	Rho guanine nucleotide exchange factor 17	1.75	0.038	Actin cytoskeleton organization
*PLEKHG6*	pleckstrin homology domain containing, family G (with RhoGef domain) member 6	1.75	0.017	
*SMARCD2*	SWI/SNF related, matrix associated, actin dependent regulator of chromatin, subfamily d, member 2	1.75	0.009	Chromatin remodeling
*SIRT2*	sirtuin 2	1.73	0.021	Cellular response to hypoxia, tubulin deacetylation, histone H4 deacetylation, histone H3 deacetylation, regulation of muscle tissue development, phosphatidylinositol-mediated signaling, regulation of angiogenesis
*AKR1B1*	aldo-keto reductase family 1, member B1 (aldose reductase)	1.72	0.049	Cellular ketone metabolic process, cellular hyperosmotic salinity response

* Biological process gene ontologies were derived using HumanBase online tool (URL: https://hb.flatironinstitute.org, accessed on 22 October 2021).

**Table 3:** Top significantly downregulated DEGs and their biological function.

Gene Symbol	Description	Fold Change	*p*-Value	Biological Process *
*SMC4*	structural maintenance of chromosomes 4	−4.89	0.019	Nuclear chromosome segregation, mitotic nuclear division, DNA conformation change, positive regulation of cell cycle process, cell cycle checkpoint, regulation of DNA repair
*ANXA5*	annexin A5	−4.71	0.023	Regulation of vesicle fusion, cellular response to TGF-β stimulus, regulation of type 2 immune response, integrin-mediated signaling pathway, apoptotic signaling pathway
*DAPP1*	dual adaptor of phosphotyrosine and 3-phosphoinositides	−4.58	0.019	Negative regulation of wound healing, cytokine-mediated signaling pathway, tyrosine phosphorylation of STAT protein, Janus kinase (JAK)-signal transducer and activator of transcription (STAT) pathway (JAK-STAT) cascade
*PHLDB2*	pleckstrin homology-like domain, family B, member 2	−4.09	0.046	Response to wounding, epithelial to mesenchymal transition, mesenchymal cell differentiation, regulation of focal adhesion assembly, negative regulation of adherens junction organization, positive regulation of apoptotic process, canonical Wnt signaling pathway, positive regulation of MAPK cascade
*SDHD*	succinate dehydrogenase complex subunit D, integral membrane protein	−3.83	0.038	Tricarboxylic acid metabolic process, drug metabolic process, energy derivation by oxidation of organic compounds, cellular respiration
*NBPF10*	neuroblastoma breakpoint family, member 10	−3.73	0.012	Negative regulation of hemopoiesis, negative regulation of cell cycle, negative regulation of cell differentiation
*RAB18*	RAB18, member RAS oncogene family	−3.61	0.041	Positive regulation of DNA-templated transcription, lipid particle organization, positive regulation of mRNA splicing
*BCLAF1*	BCL2-associated transcription factor 1	−3.47	0.004	Positive regulation of apoptotic signaling pathway, regulation of mRNA processing, posttranscriptional regulation of gene expression, DNA repair, regulation of histone modification
*HNRNPH3*	heterogeneous nuclear ribonucleoprotein H3 (2H9)	−3.44	0.019	Epithelial cell differentiation, RNA splicing, regulation of mRNA processing, regulation of translational initiation, bone morphogenetic protein (BMP) signaling pathway, positive regulation of histone modification
*TBC1D23*	TBC1 domain family, member 23	−3.4	0.013	Cytosolic transport, central nervous system development, brain development, head development
*SH3YL1*	SH3 and SYLF domain containing 1	−3.34	0.039	
*PAPD4*	PAP associated domain containing 4	−3.13	0.042	
*MIS18BP1*	MIS18 binding protein 1	−3.11	0.007	Regulation of mitotic nuclear division, positive regulation of cell cycle, cell cycle checkpoint
*SLMAP*	sarcolemma associated protein	−3.09	0.005	Regulation of action potential, membrane depolarization during cardiac muscle cell action potential, regulation of voltage-gated sodium channel activity, regulation of sprouting angiogenesis, positive regulation of stress-activated MAPK cascade
*MITD1*	microtubule interacting and trafficking domain containing 1	−3.08	0.048	Wound healing, mitotic cytokinesis, negative regulation of protein binding
*PDCD10*	programmed cell death 10	−3.06	0.043	Stress-activated MAPK cascade, response to reactive oxygen species (ROS), negative regulation of endothelial and epithelial cell proliferation, cardiovascular system development, sprouting angiogenesis, intrinsic apoptotic signaling pathway, positive regulation of Notch signaling pathway
*OSBPL8*	oxysterol binding protein-like 8	−3.05	0.009	Lipid transport, localization and storage, regulation of sequestering of triglyceride, fat cell differentiation
*SPCS2*	signal peptidase complex subunit 2	−2.99	0.043	Mitochondrial transcription, mitochondrial RNA metabolic process, purine ribonucleotide metabolic process, ATP metabolic process
*CCDC186*	coiled-coil domain containing 186	−2.98	0.01	Negative regulation of protein phosphorylation and protein modification process

* Biological process gene ontologies were derived using HumanBase online tool (URL: https://hb.flatironinstitute.org, accessed on 22 October 2021).

## Data Availability

All data generated or analyzed during this study are included in this published article and its Supplementary Information Files. Microarray data are deposited in the Gene Expression Omnibus (GEO) under Accession Number: GSE188411 (https://www.ncbi.nlm.nih.gov/geo/query/acc.cgi?acc=GSE188411/, accessed on 13 November 2021).

## Supplementary Materials

The following are available online at https://www.mdpi.com/article/10.3390/biomedicines10010048/s1, File S1: Functionally enriched modules, File S2: Tissue-specific functional enrichment, File S3: Top selected genes and their function.
